# Development of a novel model of cholecystectomy in subsequently ovariectomized mice and characterization of metabolic and gastrointestinal phenotypes: a pilot study

**DOI:** 10.1186/s12876-021-01648-1

**Published:** 2021-02-11

**Authors:** Celeste Alexander, Tzu-Wen L. Cross, Anne H. Lee, Lindsey K. Ly, Miranda D. Vieson, Jason M. Ridlon, Erik R. Nelson, Kelly S. Swanson

**Affiliations:** 1 Division of Nutritional Sciences, University of Illinois at Urbana-Champaign, 1207 W Gregory Dr, Urbana, IL 61801 USA; 2 Department of Animal Sciences, University of Illinois at Urbana-Champaign, Urbana, IL USA; 3 College of Veterinary Medicine, University of Illinois at Urbana-Champaign, Urbana, IL USA; 4 Cancer Center at Illinois, University of Illinois at Urbana-Champaign, Urbana, IL USA; 5 Carl R. Woese Institute for Genomic Biology, Anticancer Discovery from Pets to People Theme, University of Illinois Urbana-Champaign, IL Urbana, USA; 6 Department of Molecular and Integrative Physiology, University of Illinois at Urbana-Champaign, Urbana, IL USA; 7 University of Illinois Cancer Center, University of Illinois at Chicago, Chicago, IL USA

**Keywords:** Gallbladder removal, Menopause, Microbiome, Metabolism

## Abstract

**Background:**

Cholecystectomy (XGB) is the most common abdominal surgery performed in the United States and is associated with an increased post-surgery incidence of metabolic and gastrointestinal (GI) diseases. Two main risk factors for XGB are sex (female) and age (40–50 yr), corresponding with onset of menopause. Post-menopausal estrogen loss alone facilitates metabolic dysfunction, but the effects of XGB on metabolic and GI health have yet to be investigated in this population. Study objectives were to (1) identify possible short-term effects of XGB and (2) develop a novel murine model of XGB in human menopause via subsequent ovariectomy (OVX) and assess longitudinal effects of OVX on metabolism, GI physiology, and GI microbiota in XGB mice.

**Methods:**

Female C57BL/6 mice were utilized in two parallel studies (S1&S2). In S1, XGB mice were compared to a non-XGB baseline group after six wk. In S2, mice were XGB at wk0, either sham (SHM) or OVX at wk6, and sacrificed at wk12, wk18, and wk24. Body composition assessment and fresh fecal collections were conducted periodically. Serum and tissues were collected at sacrifice for metabolic and GI health endpoints.

**Results:**

Compared to baseline, XGB increased hepatic *CYP7A1* and decreased *HMGCR* relative expression, but did not influence BW, fat mass, or hepatic triglycerides after six wk. In S2, XGB/OVX mice had greater BW and fat mass than XGB/SHM. Cecal microbiota alpha diversity metrics were lower in XGB/OVX mice at wk24 compared the XGB/SHM. No consistent longitudinal patterns in fasting serum lipids, fecal microbial diversity, and GI gene expression were observed between S2 groups.

**Conclusions:**

In addition to developing a novel, clinically-representative model of XGB and subsequent OVX, our results suggest that OVX resulted in the expected phenotype to some extent, but that XGB may modify or mask some responses and requires further investigation.

## Introduction

Cholecystectomy (XGB) is the surgical removal of the gallbladder due to disease—most commonly symptomatic cholelithiasis (gallstone formation) and gallbladder cancer—and is the most common abdominal surgery performed in the United States, with over 800,000 procedures performed each year and estimated costs exceeding $6 billion annually [1, 2]. Individuals who undergo XGB are typically around 49 years of age (median), female (70.7%), Caucasian (65.5%), and overweight or obese (74.2%) [3]. Somewhat paradoxically, given that the median age corresponds to peri-menopausal or post-menopausal women, estrogen increases the likelihood of gallstone formation in preclinical models [4]. However, estrogen replacement therapy following menopause has been associated with an increased risk of XGB, possibly explaining, at least in part, the greater rate of XGB in females [5–7].

Following XGB, bile is continuously released into the duodenum as it is made by the liver, regardless of fed or fasted state, chronically exposing the small intestine to small volumes and concentrations of bile acids (BA). Although some post-XGB individuals report symptoms of impaired dietary fat digestion following surgery, studies have not shown significant impairments in fat absorption [8, 9]. Although some follow-up studies have led to the belief that XGB does not result in any long-term medical consequences [10, 11], more recent epidemiological and clinical studies suggest that XGB may increase the risk of developing diseases including metabolic syndrome [12], non-alcoholic fatty liver disease [13–17], cardiovascular disease [18], GI cancers [19–22], small intestinal bacterial overgrowth [23, 24], and bile reflux [25]. XGB may also alter the composition of the fecal microbiota in human subjects [26–29]. Similar to findings in humans, XGB mouse studies have reported an accumulation of hepatic triglycerides (TG), increased fasting serum TG, and increased very low-density lipoprotein production compared to non-XGB controls [30, 31]. Collectively, these studies suggest perturbations in metabolic and gastrointestinal (GI) health following XGB, though there is still a need for additional pre-clinical studies in appropriate animal models and prospective clinical trials to confirm these findings.

It is hypothesized that the continuous flow of bile into the GI tract after XGB not only has impacts locally, but also leads to systemic health perturbations. This is because BA act as potent signaling molecules that regulate their own production, inflammatory responses, and energy metabolism [32]. Furthermore, XGB could potentially allow either blooms of bile-intolerant bacteria in the absence of larger volumes of concentrated bile that normally curbs growth or blooms of bile-tolerant bacteria. Some colonic bacteria are also capable of altering the BA pool by metabolizing BA [33]. Theoretically, XGB may result in compositional or functional changes to the GI microbiome, which in turn alters BA pool composition and subsequent metabolic signaling.

Few studies investigating metabolic changes post-XGB have been performed in animal models to date. Despite the fact that over 70% of XGB patients are female, recent research has only utilized male animal models [2, 30, 31]. While male XGB mice may provide some insight not possible in human studies, differences in sex hormones are known to greatly influence both host metabolism and the GI microbiome [34]. The median age of individuals undergoing XGB corresponds to the onset of menopause in women [3, 35]. Because menopause is known to exacerbate metabolic dysfunction, it can be argued that studying XGB in an intact, pre-menopausal animal model would not be sufficiently representative of the main clinical population [35]. One of the most commonly used and validated models of menopause in rodents is ovariectomy (OVX), the surgical removal of the ovaries. The current lack of understanding about the impact of XGB on metabolism and GI health and microbiota in middle-aged women is of clinical concern, given the number of procedures performed each year and evidence suggesting consequent negative effects. Thus, the purpose of this study was to develop a novel model of XGB in subsequently OVX mice to more closely represent the main XGB patient population, and characterize the resulting metabolic and GI phenotypes.

## Methods

### Animals, diet, and study design

All procedures were approved by the University of Illinois Institutional Animal Care and Use Committee before the study (protocol #17040) and were performed in accordance with the United States Public Health Service Policy on Humane Care and Use of Laboratory Animals. Forty-eight eight-wk-old female C57BL/6 mice (Jackson Laboratory, Bar Harbor, ME) were used in two studies run in parallel. All mice were fed a high-fat (45% kcal from fat, HFD), low-sucrose (7% kcal) diet (Table 1) for the duration of the studies (6 or 24 wk) and housed in an environmentally-controlled facility with a 12 h light/12 h dark cycle. Mice were given ad libitum access to food and intake was measured twice weekly. Mice were given access to fresh water at all times. Body weight (BW) was measured weekly throughout the study.

**Table 1:** Ingredient and macronutrient composition of the experimental diet

Ingredients	% as-fed (g/100 g)
Casein, 80 Mesh	23.307
Lard	20.685
Corn starch	20.347
Maltodextrin 10	11.654
Sucrose	8.274
Cellulose, BW200	5.827
Corn Oil	2.913
Potassium citrate, 1 H_2_O	1.923
Dicalcium phosphate	1.515
Mineral mix S10026	1.165
Vitamin mix V10001	1.165
Calcium carbonate	0.006
l-cystine	0.003
Choline bitartrate	0.002

Macronutrient composition	% kcal
Fat	45
Carbohydrate	35
Protein	20
Gross energy (kcal/g)	4.73

**Fig. 1:**
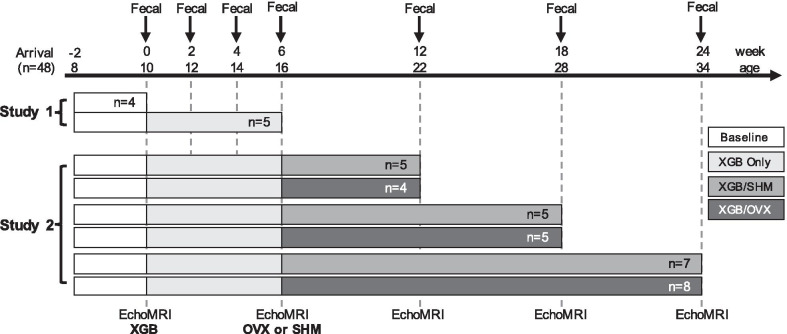
Study timelines. N-values represent the number of mice sacrificed at each time point in each group

The study timelines are presented in Fig. 1. The aim of Study 1 (S1) was to identify relatively short-term (six wk) effects of XGB on study outcomes by comparing mice to a baseline group without surgical alterations. The aim of Study 2 (S2) was to develop a novel model of XGB in OVX mice and to characterize the resulting metabolic and GI phenotypes compared to XGB mice with intact ovaries (sham OVX operation, SHM). Mice were group-housed (n = 4/cage) and adapted to the experimental diet and facilities for two wk upon arrival. To reduce variability in baseline microbiota, bedding from all cages was mixed and redistributed every 4–5 d during the adaptation period. After adaptation, mice (n = 4, ten-wk-old) were euthanized by CO_2_ inhalation for S1 baseline (wk 0) measurements and all remaining 44 mice (ten-wk-old) underwent XGB at wk 0 and were housed individually in standard shoebox cages for the remainder of the studies. In S1, mice were euthanized six wk post-XGB (n = 5) for tissue collection. In S2, mice were randomly assigned to undergo either SHM or OVX procedures on wk 6 (16-wk-old), resulting in two experimental groups: XGB/SHM and XGB/OVX. OVX was performed following XGB in order to mimic progression into menopause. Mice from each group in S2 were euthanized at wk 12, 18, and 24 for tissue collection and longitudinal analyses. In both studies, all existing mice were scanned using an EchoMRI-700 (Echo Medical Systems, Houston, TX) for body composition analysis on wk 0, 6, 12, 18, and 24. Fresh fecal samples were collected for S2 at wk 6, 12, 18, and 24 and flash frozen for microbiota analysis.

### Surgical procedures

Hair was removed at the site of operation. Mice were fasted overnight prior to surgeries. Mice were anesthetized with isoflurane (1L/min O2, 4% isoflurane reduced to 2% after anesthetized, Phoenix Pharmaceuticals, Burlingame, CA) throughout the procedures. Preparatory and surgical tasks were split between two people to maintain a sterile environment. Once under anesthesia, Puralube® (Dechra, Northwich, United Kingdom) was applied to eyes to counter-act the reduction in tearing. Mice were then injected (SQ) with 5 mg/kg carprofen (nonsteroidal anti-inflammatory, Zoetis, Parsippany-Troy Hills, NJ). The site of operation was sterilized with Povidone/Iodine swabs (Med Vet International, Mettawa, IL) and ethanol swabs three times.

#### XGB procedure

A vertical incision was made through the skin above the liver (on abdomen), followed by a vertical incision through the abdominal muscle wall. The gallbladder was externalized, emptied, and removed using cauterizing scissors. Muscle walls and skin were sutured with absorbable sutures (VCP822G, Ethicon, Somerville, NJ). Two drops of bupivacaine (0.25%, SKM Pharma Pvt Ltd., Bengaluru, India) was applied on top of the muscle wall. Medbond tissue adhesive (CP Medical, Norcross, GA) was applied to the closed incision.

#### OVX procedure

A vertical incision was made through the skin above the ovary fat pad (flank), followed by a horizontal incision through the abdominal muscle wall. The ovary was externalized and removed using cauterizing scissors. The fat pad was relocated back in the peritoneal cavity, and muscle walls were closed using absorbable sutures if needed. A drop of bupivacaine (0.25%) was applied on top of the muscle wall. Wound clips (BD Autoclip™ Wound Closing System, 9 mm, BD, Franklin Lakes, NJ) were placed on the incision. This was repeated for the other ovary. The SHM procedure was identical, except the ovaries were not removed.

After both procedures, carprofen was administered (5 mg/kg) in all mice at 24 h and 48 h post-surgery. Carprofen was administered again at 72 h only if pain or discomfort was observed. Wound clips were removed at 10–14 d post-surgery. Mice typically did not exhibit visible signs of pain or distress after 24–48 h post-surgery.

### Serum collection and analysis

Blood samples were collected immediately after euthanasia before dissection via cardiac puncture, placed in sterile microcentrifuge tubes, and allowed to clot at room temperature for 30 min prior to centrifugation for 10 min at 2000 g and 4 °C and storage at − 80 °C. Serum samples were sent to a commercial laboratory to determine TG and total cholesterol concentrations (Comparative Clinical Pathology Services, Columbia, MO) using an Olympus AU680 automated chemistry analyzer (Beckman-Coulter, Brea, CA).

### Adipose depots, hepatic lipidosis, and hepatic TG quantification

Four major adipose depots—subcutaneous, gonadal, mesenteric, perirenal—were removed at sacrifice and weighed. For pathology scoring, liver samples were fixed in 10% neutral-buffered formalin for at least 24 h then stored in 80% ethanol indefinitely. Samples were trimmed and paraffin-embedded prior to being sliced and stained with H&E at the Veterinary Diagnostic Lab at the University of Illinois. Hepatic lipidosis was scored by a blinded pathologist (MDV) at the University of Illinois. Detailed criteria for lipidosis scoring are presented in Table 2. For quantification of TG, liver samples were flash frozen upon collection and stored at − 80 °C prior to extraction. Sample extraction was adapted from previously described methods [36]. Briefly, ~ 30 mg of whole liver was added to 1 ml of chloroform–methanol (2:1 vol/vol), homogenized for 2 min using a TissueLyser LT (Qiagen, Hilden, Germany), and gently agitated overnight at 4 °C. One mL of 4 mM MgCl was added, vortexed, and centrifuged for 1 h at 1000 g at 4 °C. The organic phase was removed, evaporated overnight, and reconstituted in 500 uL tert-butanol-Triton X-114 mix (3:2 vol/vol) and vortexed. TG concentrations were then determined using a commercially available kit (#T2449 & #F6428, Sigma, St. Louis, MO) with a spectrophotometer (Synergy HT, BioTek, Winooski, VT).

**Table 2:** Hepatic lipidosis scoring criteria

Score	Criteria
0 (Normal)	< 3 macrovesicular lipid vacuoles per lobule
1 (Minimal)	5–10% hepatocytes affected, midzonal location, no hepatocyte swelling, macrovesicular lipid vacuoles
2 (Mild)	10–25% hepatocytes affected, midzonal location with some centrilobular involvement, no or mild hepatocyte swelling, mainly macrovesicular lipid vacuolation with occasional microvesicular lipid vacuolation
3 (Moderate)	25–50% hepatocytes affected, midzonal and centrilobular distribution, moderate to marked hepatocyte swelling centrilobular and midzonal; minimal to mild swelling periportal, macro- and microvesicular lipid vacuoles
4 (Marked)	50–75% hepatocytes affected, midzonal, centrilobular and some periportal distribution, moderate to severe hepatocyte swelling, macro- and microvesicular lipid vacuolation
5 (Severe)	75–95% hepatocytes affected, midzonal, centrilobular and periportal distribution, severe hepatocyte swelling centrilobular and midzonal, moderate to marked swelling periportal, macro- and microvesicular vacuolation
6 (Very Severe)	95–100% hepatocytes affected, midzonal, centrilobular and periportal distribution, severe hepatocyte swelling all zones, macro- and microvesicular vacuolation

### RNA extraction, RT-PCR, and quantitative PCR

Total RNA was extracted from liver, distal ileum, and distal colon samples using the RNeasy Mini Kit (Qiagen, Hilden, Germany). Conversion to cDNA was performed using SuperScript III Reverse Transcriptase (Invitrogen, Carlsbad, CA). Genes related to energy and cholesterol metabolism, BA synthesis, signaling, and transport, and barrier function were measured via real-time qPCR using a QuantStudio 7 Real-Time PCR System (Applied Biosystems, Foster City, CA). Genes of interest and associated primers sequences are listed in Table 3. Briefly, primers were designed and purchased from Fluidigm Deltagene Assays (Fluidigm Corporation, South San Francisco, CA) and IDT Inc. (Coralville, IA). SYBR Green PCR Master Mix (Invitrogen, Carlsbad, CA) was used for real-time amplification and detection with a reaction volume of 10 uL. The thermal protocol was 2 min at 50 °C, and 10 min at 95 °C, followed by 40 amplification cycles of 15 s at 95 °C and 1 min at 60 °C. Data analysis was performed using the QuantStudio 6 and 7 Flex Real-Time PCR System Software (Applied Biosystems, Foster City, CA). All RNA data presented were derived using the ΔΔΔΔCT method, assessing gene expression relative to the housekeeping gene (PPIA), and expressing data as fold change relative to baseline (wk 0).

**Table 3:** Target genes of interest and associated primers

Target gene (Alias)	Forward primer (5′ → 3′)	Reverse primer (5′ → 3′)
*CYP7A1*	TGTGGTAGTGAGCTGTTGCATA	GGAATCAACCCGTTGTCCAA
*FGF15*	CGCGGACGGCAAGATATAC	ACAGTCCATTTCCTCCCTGAA
*HMGCR*	ACCAAACCCCGTAACCCAAA	AGCGACTATGAGCGTGAACA
*MGLL*	GTCAATGCAGACGGACAGTAC	CATAACGGCCACAGTGTTCC
*MUC2*	CAGCACACCAACCAAAACCA	CACAGCCACCAGGTCTCATTA
*NR1H4 *(*FXR*)	GCCTCTGGGTACCACTACAA	GTACACGGCGTTCTTGGTAA
*SLC10A1 *(*NTCP*)	TTGCGCCATAGGGATCTTCC	TGATGACAGACAGGACTGTGAC
*SLC10A2 *(*ASBT*)	TGGAGGAACTGGCTCCAATA	GGAGCAAGTGGTCATGCTAA

### Microbiota analysis

Cecal and fecal bacterial DNA from S2 was extracted using the PowerLyzer PowerSoil Kit (Qiagen, Hilden, Germany) with bead beating using a vortex adaptor (cat. no. 13000-V1-24, Qiagen, Hilden Germany). The concentration of extracted DNA was quantified using a Qubit® 3.0 Fluorometer (Life Technologies, Carlsbad, CA). 16S rRNA gene amplicons of the V4 region were generated, pooled, and sequenced for each sample as previously described by our lab [37]. 16S rRNA gene amplicons were generated using a Fluidigm Access Array (Fluidigm Corporation, South San Francisco, CA) in combination with Roche High Fidelity Fast Start Kit (Roche, Basel, Switzerland). The primers 515F (5′-GTGYCAGCMGCCGCGGTAA-3′) and 806R (5′-GGACTACNVGGGTWTCTAAT-3′) that target a 252-bp fragment of the V4 region were used for amplification (primers synthesized by IDT Inc., Coralville, IA) [38]. CS1 forward tag and CS2 reverse tag were added according to the Fluidigm protocol. Quality of the amplicons was assessed using a Fragment Analyzer (Advanced Analytics, Ames, IA) to confirm amplicon regions and sizes. A DNA pool was generated by combining equimolar amounts of the amplicons from each sample. The pooled samples were then size-selected on a 2% agarose E-gel (Life Technologies, Carlsbad, CA) and extracted using a Qiagen gel purification kit (Qiagen, Hilden, Germany). Cleaned size-selected pooled products were run on an Agilent 2100 Bioanalyzer to confirm appropriate profile and average size. Sequencing was performed at the W. M. Keck Center for Biotechnology at the University of Illinois using an Illumina MiSeq using version 3 reagents (Illumina Inc., San Diego, CA).

Forward reads were trimmed using the FASTX-Toolkit (version 0.0.14) and QIIME 2.2019.4 was used to process the resulting sequence data [39]. An average of 38,896 reads per sample were obtained before processing. Briefly, high-quality (quality value ≥ 20) sequence data derived from the sequencing process were demultiplexed. Data were then denoised and assembled into a feature table of amplicon sequence variants (ASV) using DADA2 [40]. Taxonomy was assigned using the SILVA 132 database [41]. An even sampling depth of 15,802 and 10,123 sequences per sample was used for assessing alpha- and beta-diversity measures for cecal and fecal samples, respectively. Alpha-diversity was estimated using observed-OTUs, Shannon’s index, and Faith’s PD metrics. Beta-diversity was assessed using weighted and unweighted UniFrac distance measures and presented with principal coordinates analysis (PCoA) plots [42].

### Statistical analysis

Repeated measures data (S2 body weight and EchoMRI) were analyzed using the ‘glimmix’ procedure for repeated measures data, testing the main effects and interaction of surgery and time in SAS (version 9.4; SAS Institute). Data collected at the time of sacrifice were analyzed using either the exact Wilcoxon signed-rank test (proc npar1way) or Student’s t test (proc ttest) in SAS. For microbiota sequencing data, assigned taxa that failed to reach an average relative abundance of at least 0.01% across all samples or were not detectable in at least 20% of all samples were discarded. Statistical significance was set as *p* < 0.05 with *p* < 0.10 considered as trends. Data are either represented as boxplots or reported as least square means with standard errors of the mean (SEM).

## Results

**Fig. 2:**
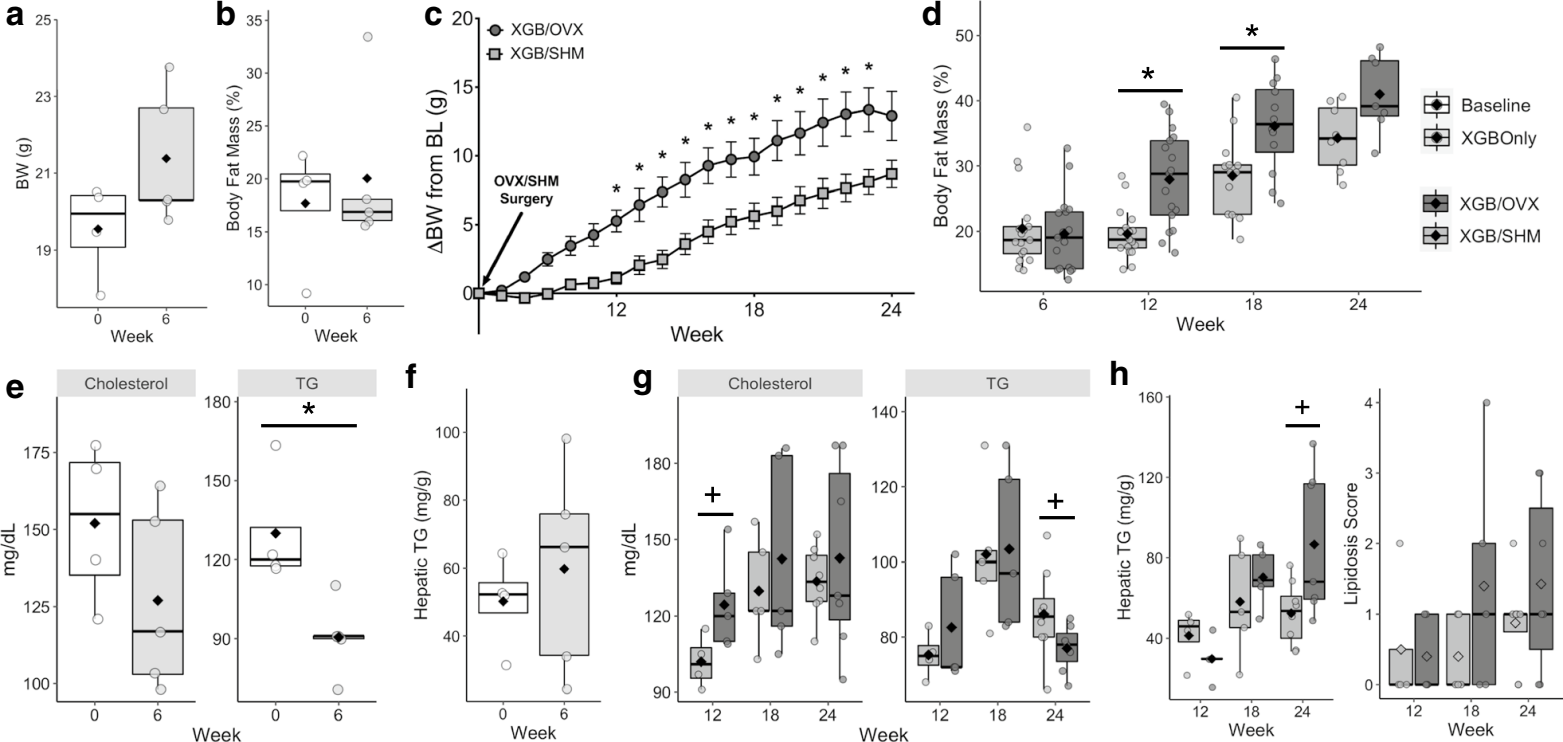
Body weight (BW) (**a**, **c**), fat mass (**b**, **d**), blood lipids (**e**, **g**), and hepatic steatosis (**f**, **h**) of mice undergoing cholecystectomy (XGB) only or XGB followed by either a sham (SHM) or ovariectomy (OVX) operation. In **a**, **b**, **e**, **f** means are represented by a diamond, wk 0, n = 4; wk 6, n = 5. Data in panel **c** are expressed as LS means ± SEM. In **c**, **d** wk 6–12, n = 17 per group; wk 13–18, n = 12–13 per group; wk 19–24, n = 7–8 per group. In **g**, **h** means are represented by a diamond, wk 12, n = 4–5 per group; wk 18, n = 5 per group; wk 24, n = 7–8 per group. Main effects of surgery are denoted by *(*p* < 0.05). Trends are denoted by +(*p* < 0.10). *TG* triglycerides

**Fig. 3:**
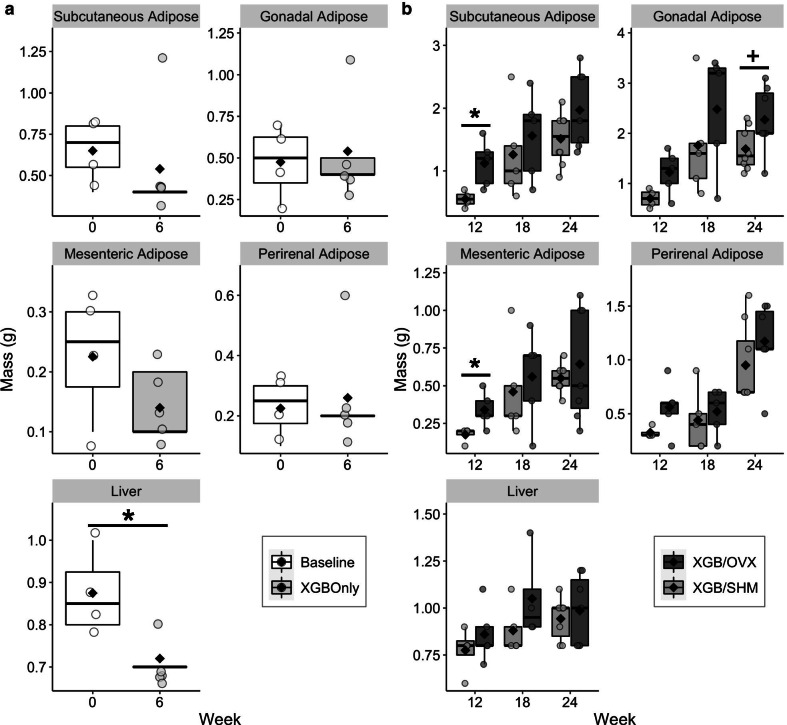
Adipose depot and liver tissue masses of mice undergoing cholecystectomy (XGB) only (**a**) or XGB followed by either a sham (SHM) or ovariectomy (OVX) operation (**b**). In **a** wk 0, n = 4; wk 6, n = 5. In **b** wk 12, n = 4–5 per group; wk 18, n = 5 per group; wk 24, n = 7–8 per group. Means are represented by a diamond. Main effects of surgery are denoted by *(*p* < 0.05). Trends are denoted by +(*p* < 0.10)

Within six wk, XGB did not result in differences in BW (Fig. 2a) or body fat mass (Fig. 2b) compared to baseline. Over time, XGB/OVX led to greater (*p* < 0.001) weight gain compared to XGB/SHM, with groups being statistically different from one another from wk 12 to wk 23 (Fig. 2c). Body fat mass was also greater (*p* < 0.05) in XGB/OVX mice than XGB/SHM mice at wk 12 and 18 (Fig. 2d). In S1, XGB did not result in any differences in mass of major adipose depots; however, total liver mass was reduced (*p* = 0.03) compared to baseline (Fig. 3a). In S2, XGB/OVX mice exhibited greater subcutaneous (*p* = 0.02) and mesenteric (*p* = 0.04) adipose depot masses compared to XGB/SHM mice at wk 12, but not wk 18 or 24 (Fig. 3b). XGB resulted in reduced (*p* = 0.013) fasting serum TG concentrations after six wk compared to baseline in S1, but no differences in serum total cholesterol concentrations were observed (Fig. 2e). Liver TG content was not altered six wk post-XGB (Fig. 2f). No consistent longitudinal patterns were observed in serum total cholesterol and TG concentrations between groups in S2 (Fig. 2g), likely due to a high degree of variation. At wk 12, serum total cholesterol concentrations tended to be greater in XGB/OVX compared to XGB/SHM (*p* = 0.068), but were not different at wk 18 and 24. Additionally, serum TG concentrations tended (*p* = 0.099) to be lower in XGB/OVX compared to XGB/SHM at wk 24, but not other timepoints. At wk 24, XGB/OVX mice tended to have greater hepatic TG content compared to XGB/SHM; however, hepatic lipidosis scoring revealed no differences between groups (Fig. 2h).

**Fig. 4:**
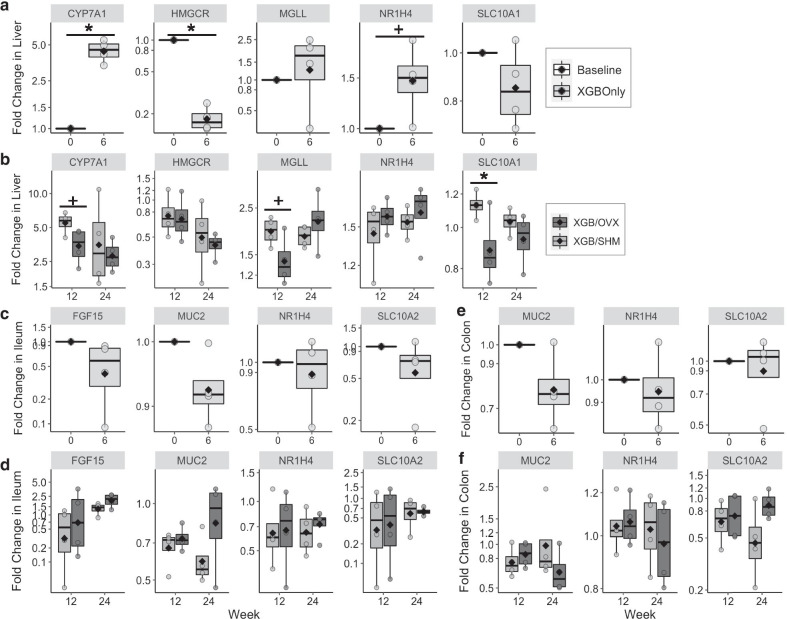
Relative gene expression in liver (**a**, **b**), ileal (**c**, **d**), and colonic (**e**, **f**) tissue of mice undergoing cholecystectomy (XGB) only or XGB followed by either a sham (SHM) or ovariectomy (OVX) operation. Data are expressed as fold change in expression relative to baseline (wk 0), n = 4 per group in all panels. Means are represented by a diamond. Main effects of surgery are denoted by *(*p* < 0.05). Trends are denoted by +(*p* < 0.10). CYP7A1, cholesterol 7 alpha-hydroxylase; HMGCR, HMG-CoA reductase; MGLL, monoacylglycerol lipase; NR1H4, farnesoid X receptor; SLC10A1, Na^+^-taurocholate cotransporting polypeptide; FGF15, fibroblast growth factor 15; SLC10A2, apical sodium-dependent bile acid transporter; MUC2, mucin 2

Expression of genes associated with BA signaling, synthesis, and transport, barrier function, and lipid metabolism were assessed in liver, ileal, and colonic tissues. In S1, hepatic relative expression of *CYP7A1* (BA synthesis) was increased almost fivefold (*p* = 0.003), while relative expression of *HMGCR* (cholesterol synthesis) was reduced more than fivefold (*p* = 0.0002) in XGB mice compared to baseline (Fig. 4a). Additionally, relative expression of *NR1H4* (FXR, major BA receptor) tended to be 1.5-fold greater (*p* = 0.058) within six wk post-XGB compared to baseline. No differences were observed between XGB and baseline mice in the relative expression of hepatic *MGLL* (monoacylglyceride catabolism) or *SLC10A1* (BA reabsorption). No differences in relative gene expression of *FGF15* (FGF19 ortholog, feedback inhibition of BA synthesis, energy homeostasis), *MUC2* (intestinal mucin), *NR1H4*, or *SLC10A2* (BA reabsorption) were observed in ileal tissues (Fig. 4c) between baseline and six wk post-XGB. Additionally, no differences in relative gene expression of *MUC2*, *NR1H4*, or *SLC10A2* were observed in colonic tissues (Fig. 4e) between baseline and six wk post-XGB.

In liver tissues of S2, relative expression of *SLC10A1* was lower (*p* = 0.048) in XGB/OVX mice compared to XGB/SHM at wk 12 but not different at wk 24 (Fig. 4b). Relative expression of *CYP7A1* (*p* = 0.07) and *MGLL* (*p* = 0.08) tended to be lower in XGB/OVX mice compared to XGB/SHM at wk 12, but were not different at wk 24. Relative expression of hepatic *CYP7A1* and *NR1H4* appeared to remain elevated and *HMGCR* appeared to remain reduced in all S2 mice compared to S1 baseline mice, suggesting potentially chronic effects of XGB (Fig. 4). Similar to S1, there were no differences in relative gene expression observed in ileal or colonic tissues (Fig. 4d, f) between XGB/OVX and XGB/SHM mice at either time point.

**Fig. 5:**
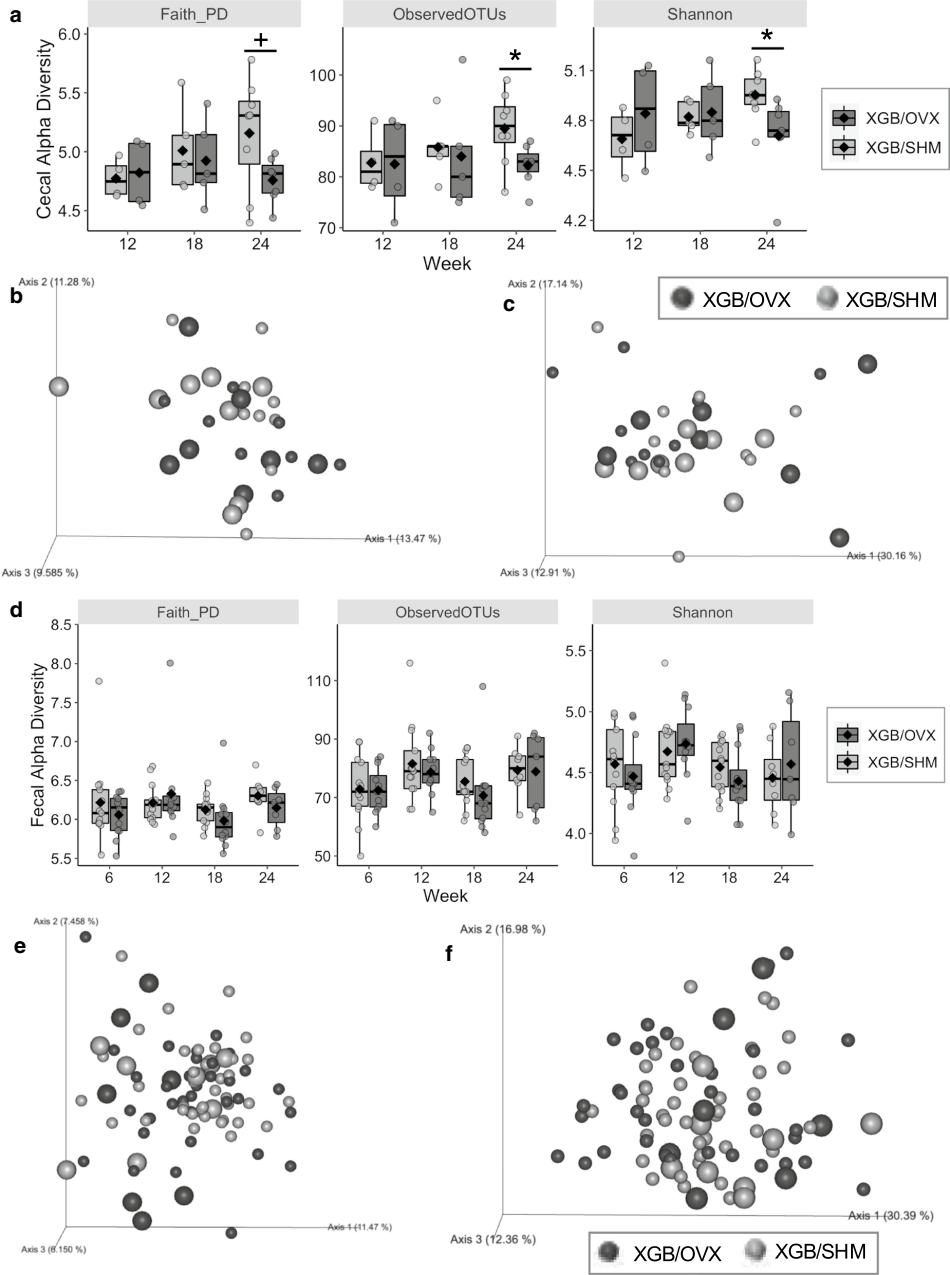
Alpha (**a**, **d**) and beta (**b**, **c**, **e**, **f**) diversity metrics of cecal (**a**–**c**) and fecal (**d**–**f**) microbiota of mice undergoing cholecystectomy (XGB) followed by either a sham (SHM) or ovariectomy (OVX) operation. Means in **a**, **c** are represented by a diamond. In **a**–**c**, wk 12, n = 4–5 per group; wk 18, n = 5 per group; wk 24, n = 7–8 per group. In **d**–**f**, n = 7–13 per group. **b**, **e** unweighted Unifrac distance principal coordinate analysis. **c**, **f** weighted Unifrac distance principal coordinate analysis. In **b**, **c**, **e**, **f**, each dot represents a single sample. Larger dots are specific to wk 24. Main effects of surgery are denoted by *(*p* < 0.05). Trends are denoted by +(*p* < 0.10)

**Fig. 6:**
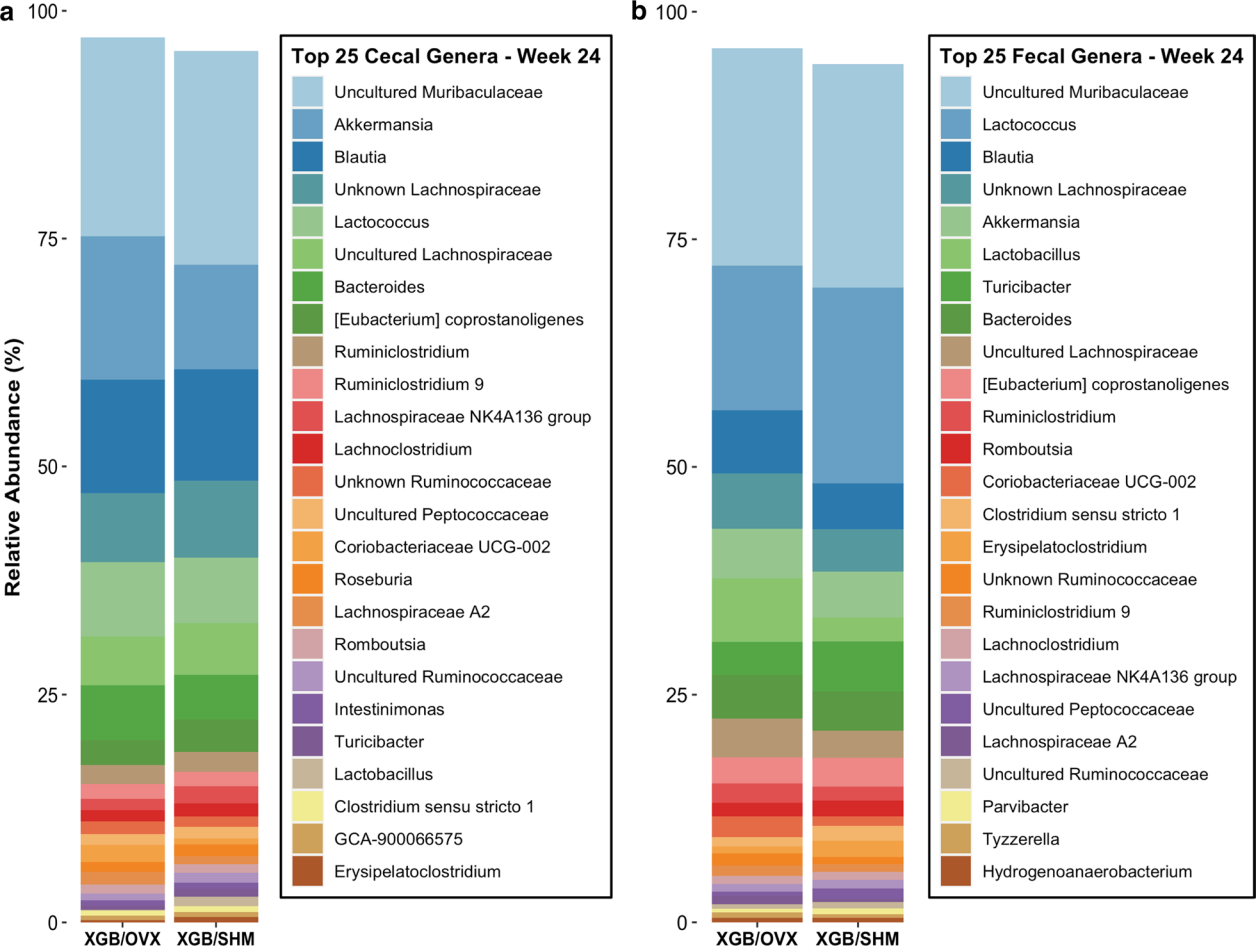
Most abundant microbial genera at wk 24 in cecal and fecal samples of mice undergoing cholecystectomy (XGB) and followed by either a sham (SHM) or ovariectomy (OVX) operation. Data are expressed as means. In both samples, n = 7–8 per group

**Fig. 7:**
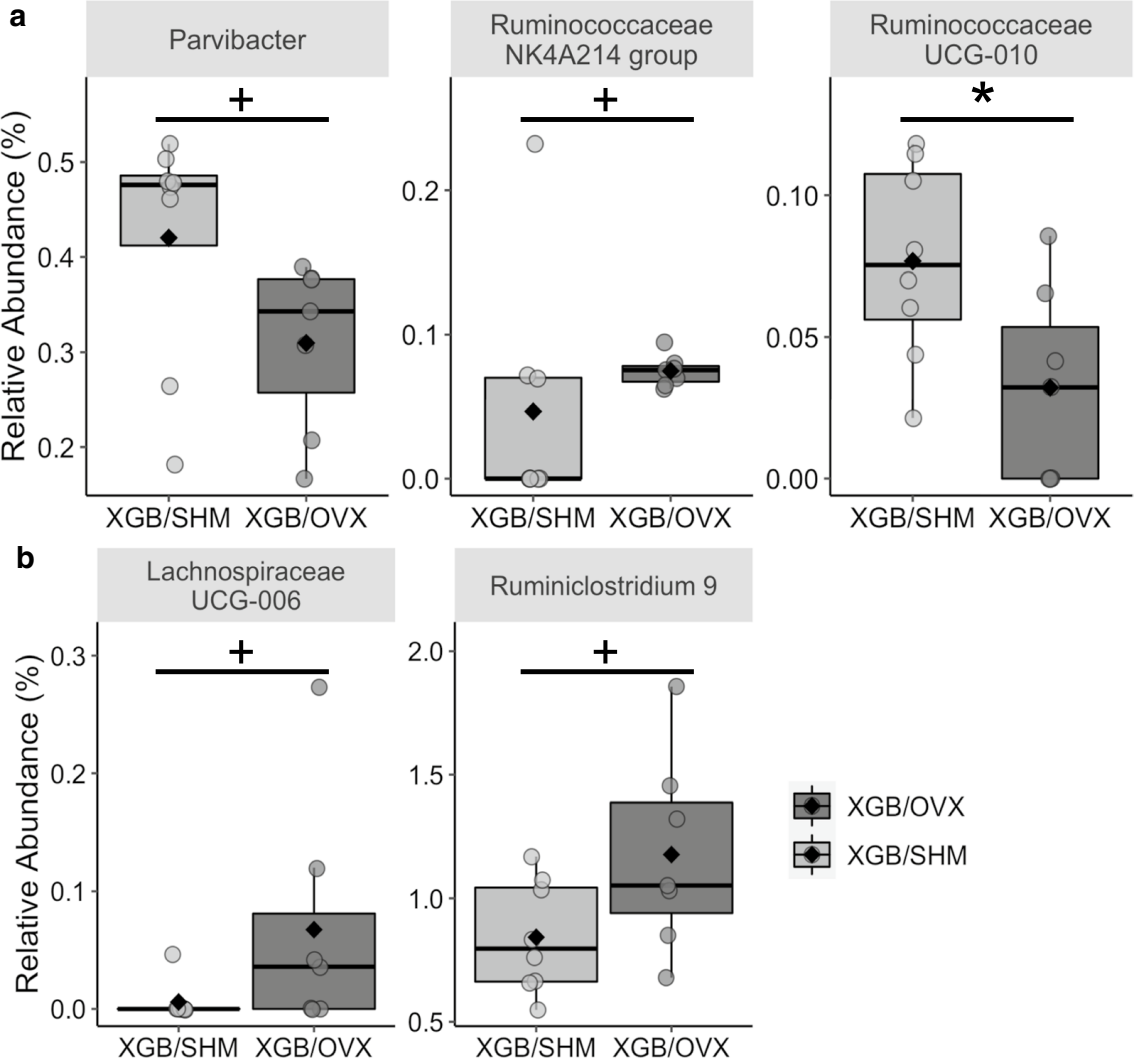
Differentially abundant taxa of cecal (**a**) and fecal (**b**) samples at wk 24 of mice undergoing cholecystectomy (XGB) followed by either a sham (SHM) or ovariectomy (OVX) operation. In all panels, n = 7–8 per group. Means are represented by a diamond. Main effects of surgery are denoted by *(*p* < 0.05). Trends are denoted by +(*p* < 0.10)

Diversity and composition of cecal and fecal microbial samples were assessed in S2 mice. Cecal microbial OTU richness (*p* = 0.03) and Shannon’s diversity index (*p* = 0.03) were lower in XGB/OVX mice at wk 24 compared to XGB/SHM (Fig. 5a). Faith’s phylogenetic diversity tended (*p* = 0.058) to be lower in XGB/OVX mice at wk 24 compared to XGB/SHM. No differences in cecal beta diversity as measured by unweighted (Fig. 5b) or weighted (Fig. 5c) Unifrac distances were observed between XGB/OVX and XGB/SHM mice at any time point, and no distinct clustering was observed in PCoA plots. In fecal samples, no differences in any alpha diversity metrics were observed between XGB/OVX and XGB/SHM mice at any time point (Fig. 5d). Similarly, no differences in beta diversity as measured by unweighted (Fig. 5e) or weighted (Fig. 5f) Unifrac distances were observed. Visualization of the 25 most abundant genera in both cecal and fecal samples demonstrated similar compositions between XGB/OVX and XGB/SHM mice at the end of S2 (Fig. 6). A few individual genera were observed to be differentially abundant. In cecal samples, relative abundance of *Ruminococcaceae UCG-010* was lower (*p* = 0.03) in XGB/OVX compared to XGB/SHM mice (Fig. 7a). Additionally, relative abundance of cecal *Parvibacter* tended (*p* = 0.07) to be lower and *Ruminococcaceae NK4A214 group* tended (*p* = 0.07) to be greater in XGB/OVX compared to XGB/SHM mice. In fecal samples, XGB/OVX mice tended to have greater relative abundances of *Lachnospiraceae UCG-006* (*p* = 0.08) and *Ruminiclostridium 9* (*p* = 0.06) compared to XGB/SHM mice (Fig. 7b).

## Discussion

In clinical trials, XGB has been shown to result in increased weight gain within six mo post-surgery [43]. Interestingly, within 3 yr of XGB, females, but not males, were reported to have increased BMI compared to pre-op [44]. Pre-clinical XGB studies with male mice have not reported increased weight gain compared to SHM controls, suggesting there may be sex-specific responses to surgery [30, 31, 45]. In the present study, no changes in BW, body fat mass, or adipose depot masses were observed within six wk of XGB compared to baseline, but a non-XGB age-matched control was not utilized. Although clinical research has demonstrated an association between XGB and hepatic steatosis, no difference in hepatic TG was observed between XGB and baseline mice in this study [13–17]. Development of steatosis due to XGB may take longer than six wk, thus further preclinical research in females utilizing age-matched, intact-gallbladder controls is needed.

Human menopause is associated with increased weight gain, ectopic fat deposition, and increased incidence of metabolic syndrome [35]. The rodent OVX model results in a similar phenotype. Previous studies in OVX rats and mice have demonstrated an increase in BW, fat mass, and hepatic TG content and lipidosis compared to intact controls [46–49]. However, it is not known if OVX results in a similar phenotype in XGB mice as in those with an intact gallbladder. A greater gain in total BW, particularly body fat mass, was observed in XGB/OVX mice compared to XGB/SHM mice. Increased fat mass tended to be predominantly in gonadal fat pads by wk 24, which is consistent with previous findings [49]. Interestingly, the difference in BW gain between XGB/OVX and XGB/SHM groups appears to plateau around wk 14–15, potentially suggesting a delayed onset of rapid weight gain due to XGB that is accelerated by OVX. Hepatic TG only tended to be higher in XGB/OVX compared to XGB/SHM by the end of the study. Over time, hepatic TG content increased in all S2 mice, which was likely due to a combination of aging and long-term consumption of a HFD, but potentially influenced by the XGB procedures at baseline. Previous studies in OVX mice and rats have also shown that OVX results in increased fasting LDL concentrations, with no consistent changes in fasting total cholesterol or TG observed [46, 49]. The present results were consistent with these findings in that no distinct patterns were observed in fasting total cholesterol or TG concentrations over time, however, LDL was not measured. In the present study, OVX was performed following XGB to mimic progression into menopause. Future studies would be useful to determine if timing of OVX, before or after XGB, differentially effects these outcomes.

Interestingly, there were no observed differences in relative expression of genes related to energy metabolism, gut integrity, or BA metabolism and transport in the ileum or colon between groups in both S1 or S2. In S1, hepatic relative expression of *CYP7A1* was increased almost fivefold while relative expression of *HMGCR* was decreased more than fivefold, suggesting a shift away from endogenous cholesterol synthesis toward BA synthesis. In contrast, previous research in male XGB mice has not measured *HMGCR* expression, but reported that hepatic *CYP7A1* relative expression either does not change two-wk post-XGB [2] or decreases five-wk post-XGB [30] compared to SHM controls. While the effects in the present study could be influenced by aging, the response is likely to be, at least in part, due to XGB and/or chronic HFD consumption, as these genes encode proteins involved in lipid and BA metabolism. In non-XGB C57BL/6 mice, females are reported to have greater hepatic *CYP7A1* expression compared to males [50]. Sexually dimorphic expression of *CYP7A1* prior to XGB may explain contrasting results with previous research. Additionally, previous research demonstrated that female C57BL/6 mice fed a HFD (60% kcal) had dramatically increased hepatic *CYP7A1* expression and no change in *HMGCR* expression compared to low-FD (16% kcal) controls after 9 wk [51]. In contrast again with the present findings, a study in male mice demonstrated that after five-wk post-XGB, ileal, but not hepatic, relative expression of *NR1H4* was increased compared to controls. These findings collectively suggest there may be sex-specific effects of XGB on host health that have yet to be elucidated, and reinforce the critical need for detailed research in female XGB mice. Previous work from our lab has demonstrated that OVX in C57BL6 mice results in a number of changes in gene expression, including an increase in hepatic expression of *MGLL* and a decrease in cecal expression of *NR1H4* and *FGF15* (unpublished). Such findings were not observed in S2—XGB/OVX did not influence relative expression of *NR1H4* in any tissues compared to XGB/SHM. Relative expression of hepatic *CYP7A1*, *MGLL*, and *SLC10A1* tended to be lower in XGB/OVX mice at wk 12, but this effect was no longer observed at wk 24. XGB may influence the expression of similar pathways, potentially masking differences due to OVX.

The present study did not observe any substantial effects of OVX on the diversity or composition of the cecal or fecal microbiota in XGB mice. By the end of the study, cecal alpha diversity metrics were lower in XGB/OVX mice, but no effect on beta diversity was observed. While one study has demonstrated that OVX may affect beta diversity [52], previous studies in our lab suggest no effect of OVX on alpha or beta diversity in mice fed a HFD or in rats fed a LFD (45, and unpublished). Additionally, there were no dramatic differences in relative abundance of individual genera in cecal and fecal samples. A few genera were found to be differentially abundant, specifically *Ruminococcaceae UCG-010*, *Parvibacter*, *Ruminococcaceae NK4A214 group*, *Lachnospiraceae UCG-006*, and *Ruminiclostridium 9*, but the physiological significance of these shifts is unknown. In contrast, previous studies in rats and mice have shown that OVX may increase relative abundance of *Akkermansia*, *Anaerovibrio*, *Bifidobacterium animalis*, *Desulfovibrio*, *Dorea*, and *Lactobacillus*, and decrease relative abundance of *Ruminococcus* and *SMB53* [46, 52]. It is possible that baseline XGB may influence the microbiota composition and mask the effects of OVX. More research is needed to understand the independent effects of XGB on the microbiome.

## Conclusions

In summary, we developed and demonstrated the feasibility of a novel model of XGB and subsequent OVX to best represent the main clinical population undergoing cholecystectomy. Furthermore, our pilot study demonstrated that in some ways, as in increased BW and fat mass, OVX resulted in the expected phenotype in previously XGB mice. However, the lack of other previously reported changes may be due to small sample size or potential metabolic alterations due to XGB. Future research utilizing our model is needed to elucidate the independent effects of XGB in female mice due to its translational and clinical potential.

## Data Availability

Datasets used in the present study are available from the corresponding authors upon request.

## Abbreviations

BA: Bile acid
BW: Body weight
d: Day
GI: Gastrointestinal
mo: Month
OVX: Ovariectomy
PCoA: Principal coordinate analysis
SEM: Standard error of the mean
SHM: Sham
SQ: Subcutaneous
TG: Triglyceride
wk: Week
XGB: Cholecystectomy
y: Year
